# Highly Effective *Ex Vivo* Gene Manipulation to Study Kidney Development Using Self-Complementary Adenoassociated Viruses

**DOI:** 10.1155/2014/682189

**Published:** 2014-07-14

**Authors:** Tie-Lin Chen, Hong-Lian Wang, Yun-Hong Liu, Yin Fang, Rui-Zhi Tan, Pu-Hui Zhou, Qin Zhou, Xiao-Yan Lv

**Affiliations:** ^1^Core Facility of Genetically Engineered Mice, State Key Laboratory of Biotherapy and Cancer Center, West China Hospital, Sichuan University, Chengdu 610041, China; ^2^College of Laboratory Medicine, Chongqing Medical University, Chongqing 400016, China; ^3^Departments of Dermatology, West China Hospital, Sichuan University, Sichuan 610041, China

## Abstract

*Background*. *Ex vivo* culture of intact embryonic kidney has become a powerful system for studying renal development. However, few methods have been available for gene manipulation and have impeded the identification and investigation of genes in this developmental process. *Results*. Here we systemically compared eight different serotypes of pseudotyped self-complementary adenoassociated viruses (scAAVs) transduction in cultured embryonic kidney with a modified culture procedure. We demonstrated that scAAV was highly effective in delivering genes into and expressing in compacted tissues. scAAV serotypes 2 and 8 exhibited higher efficiency of transduction compared to others. Expression kinetics assay revealed that scAAV can be used for gene manipulation at the study of UB branching and nephrogenesis. Repressing WT1 in cultured kidney using shRNA impairs tubule formation. We for the first time employed and validated scAAV as a gene delivery tool in cultured kidney. *Conclusions*. These findings are expected to expedite the use of the *ex vivo* embryonic kidney cultures for kidney development research. For other *ex vivo* cultured organ models, scAAV could also be a promising tool for organogenesis study.

## 1. Introduction

*Ex vivo *intact organ cultures have become an excellent model for analysing normal and impaired organogenesis. As one of the best* ex vivo* models in the study of renal development,* ex vivo* embryonic kidney cultures were developed in 1960s and have been of great value in application [1–3]. They could faithfully recapitulate many aspects of early renal development [4]. Inhibitory drugs, exogenous growth factors, function-blocking antibodies, vitamins, oligosaccharides, antisense oligonucleotides, short interfering RNAs, and recombinant transduction protein have been used to study the developmental functions of specific genes or factors [5, 6]. One of the standard methods for kidney development study is to perform gain-of-function or loss-of-function assay of the target gene and to observe associated phenotype changes. Due to the three-dimensional nature of kidney, there are limited methods of* ex vivo *gene manipulation that are capable of penetrating into the cultured organs, for example, the condensed cap mesenchyme, which will undergo mesenchyme-to-epithelial conversion and generate renal vesicle, comma- and S-shaped bodies, podocytes, and renal tubule compartments of the mature nephron [7–9].

HIV TAT-mediated protein transduction [10], siRNA transfection [11], plasmid microinjection, and electroporation [12] methods have been devised to achieve gene manipulation in cultured kidney. However, their limitations are quite evident. Protein transduction based on TAT, a protein transduction peptide which can mediate cellular uptake of fused proteins, requires production and purification of fusion proteins in* Escherichia coli* [13]. siRNA transfection shows uneven penetration and fails to enter the condensed cap mesenchyme [14]. Plasmid microinjection and electroporation only allows gene expression at the site of delivery and may cause cell damage after electroporation. Therefore, new methods of gene manipulation in cultured kidney need to be developed eagerly.

Viral vectors, such as* Lentivirus*, adenovirus and adenoassociated virus (AAV), are tools commonly used to deliver genetic materials into cells. Among nonviral and viral vectors, the small DNA virus AAV is a promising tool for gene therapy, due to its capability to cross the blood-brain barrier and deliver gene in the neonatal mouse central nervous system [15–17]. Self-complementary adenoassociated virus (scAAV) overcomes the primary barrier of complementary-strand DNA synthesis of wild-type AAV and offers faster gene expression and higher transduction efficiency. It utilizes the tendency of packaging DNA dimers when the viral genome is half length of wild-type AAV and bears a mutated inverted terminal repeat (ITR) [18, 19]. The properties of scAAV, such as high efficiency of transduction, early onset of gene expression, small size of virus, and high penetrability, make it a promising tool for kidney development study. Although AAV has been used to deliver gene in adult rat and cat kidney* in vivo *for gene therapy [20–22], there is no report about scAAV in the study of kidney development or transduction in cultured kidney. Here we tested eight different serotypes of pseudotyped scAAVs transduction in* ex vivo *cultured intact mouse kidney with a modified culture procedure and found that serotypes 2 and 8 of scAAV exhibit the highest efficiency of penetration. The utility value of scAAV was validated by scAAV2/8 carrying shRNA of WT1. Our results show that scAAV is a highly effective tool for delivering gene into* ex vivo* cultured intact embryonic kidneys.

## 2. Materials and Methods

### 2.1. Animals

C57BL/6J were purchased form The Jackson Laboratory (Bar Harbor, ME) and maintained in a specific pathogen-free facility. Pregnant mice were obtained by natural mating and were timed from the day of the vaginal plug which was designated as embryonic day 0.5 (E0.5). At day 11 to 15, pregnant mice were sacrificed for kidney isolation. All procedures were complied with the guidelines of the Institutional Animal Care and Use Committee at Sichuan University.

### 2.2. Cell and Embryonic Kidney Culture

MK3 and HEK 293T cells were cultured in Dulbecco's modified Eagle medium (DMEM, Gibco, Grand Island, NY) with 10% FBS. The cells were incubated at 37°C in a humidified atmosphere of 5% CO_2_. Kidney rudiments were microdissected under sterile conditions from timed-pregnant embryonic day 12.5 to 15.5 C57BL/6J mouse embryos. Embryonic staging was verified using the criteria of Theiler. The kidneys were placed at middle of the bottom of 8 central wells in 24-well plate. The wells were added 150 uL DMEM plus 10% FBS and the interspaces between wells were filled with PBS to reduce the evaporation of medium. All cultures were incubated at 5% CO_2_ at 37°C, half volume of medium being changed every day.

### 2.3. siRNA Transfection

Kidney rudiments were microdissected from E12.5 C57BL/6J mouse embryos and then pooled and assigned randomly to different experimental groups. Transfections were as detailed in the Lipofectamine 2000 protocol, using 50 pmol of siRNA and 5 uL Lipofectamine 2000. The 100 *μ*L transfection mix was added into 400 *μ*L DMEM plus 2% FBS in 1.5 mL tube and kept at 4°C for 6–8 hours.

### 2.4. *Lentivirus*, Adenovirus, and scAAV Transduction

Three to five kidney rudiments were incubated with 1 × 10E8 transducing units (TU, tested in HEK293T cells) per mL* Lentivirus* or adenovirus, 2 × 10E11 particles per mL scAAV in DMEM (Gibco, Grand Island, NY) supplemented with 2% FBS (MingHai Bio, Lanzhou, China) at 4°C for 6–8 hours. The ready-to-use* Lentivirus* and adenovirus are gifts from Dr. Xianming Mo (Sichuan University, Sichuan, China) and the ready-to-use different serotypes of pseudotyped scAAVs are gifts from Dr. Gao (University of Massachusetts Medical School, Worcester, MA). shRNA was cloned into pdsAAV-CB-U6-EGFP vector and targeted to mouse WT1 (AACCAAGGATACAGCACGGTC).

### 2.5. Western Blot

After incubation, the cells were washed two times with PBS and lysed by RIPA lysis buffer (Beyotime, Jiangsu, China) immediately and then placed on ice for 30 min. Total protein of each extract was boiled with SDS-PAGE loading buffer at 100°C for 5 min and loaded into 10% SDS-PAGE gel and subsequently electrotransferred to PVDF membrane (Millipore, Bedford, MA). The membranes were blocked with 5% fat-free milk in Tris-buffered saline (TBS)/Tween 20 for 0.5 h. Primary antibodies specifically against WT1 (Santa Cruz Biotech, Paso Robles, CA) and beta-tubulin (Zhongshan Bio, Beijing, China) were incubated overnight at 4°C in 5% BSA with TBS/Tween 20. Washed membranes and incubated for 1 h at room temperature with horseradish peroxidase-linked anti-mouse/rabbit IgG (1 : 1000, Santa Cruz Biotech, Paso Robles, CA) which were prepared in blocking solution. After washing, the Western Blot Luminol Reagent (Zhongshan Bio, Beijing, China) was applied for antibody detection with X-ray film.

### 2.6. Immunostaining

The cultured kidney rudiments or 15 um frozen sections were fixed in 4% PFA in PBS for 30 min at room temperature, washed with PBS 10 min for 3 times, and then incubated with primary antibodies at 4°C overnight in blocking buffer (PBS plus 1% BSA and 0.4% Triton-X-100). The primary antibodies are rabbit anti-laminin (1/200 dilution; Boster, Wuhan, China), rabbit anti-E cadherin (1/300 dilution, Abcam, Cambridge, MA), rabbit anti-WT1 C-19 (1/100, Santa Cruz Biotech, Paso Robles, CA), and mouse anti-GFP Clone 3E6 (Figures 3 and 4 and Figures S2 and S3) (see Figures S2 and S3 in the Supplementary Material available online at http://dx.doi.org/10.1155/2014/682189) (1/100 dilution; MP Biomedicals, Santa Ana, CA). Samples were washed in PBS for at least 3∗10 min and incubated with appropriate secondary antibodies in blocking buffer overnight at 4°C. The secondary antibodies were goat anti-mouse Alexa Fluor 488 and goat anti-rabbit Alexa Fluor 555 (1/100 dilution; Invitrogen, Grand Island, NY). After another 3∗10 min wash in PBS and 30 min incubation with 0.25 ug/mL DAPI in PBS, the kidney was mounted with Fluoromount-G (SouthernBiotech, New Orleans, LA) and sealed between coverslips using nail varnish. The pictures were captured with Ziess Axiovert 200 M microscope or LSM 510 confocal microscope and analyzed with NIH ImageJ 1.47a.

### 2.7. Statistical Analysis

The results are presented as the mean ± standard error of the mean (SEM). Statistical analyses were performed with the Prism 5 (GraphPad, San Diego, CA). The average fluorescence intensity was calculated by analysis of five areas (200 × 200 *μ*m) in each of the transducted kidneys with ImageJ 1.47a. The data was analyzed by Student's* t*-test. Values of *P* < 0.05 were considered as statistically significant.

## 3. Results

### 3.1. Viral Transduction with a Modified Culture Procedure

**Figure 1:**
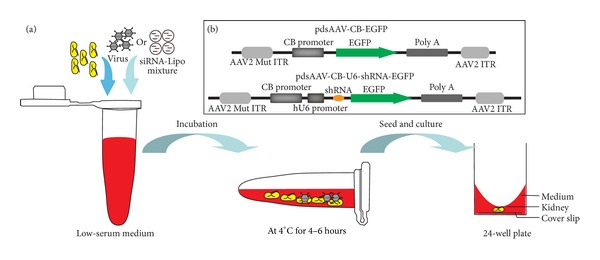
Diagram of the modified culture procedure and scAAV constructs used in this study. (a) Diagram of the modified culture procedure, in which kidney rudiments were incubated with transfection mixture or virus in 1.5 mL tube with low-serum medium for 4–6 hours at 4°C and then seeded and cultured on cover slip in 24-well plate. (b) Diagram of packaging vector containing AAV2-ITRs, CB promoter-driven EGFP transgene, and/or U6 promoter-driven shRNA. These vectors were packaged within different serotypes of capsids.

Conventional and the low-volume culture method both support the kidney rudiment at the air-medium interface, with only a thin film of medium covering it [5], which is unfit for transfection and transduction. To allow penetration of virus and transfection mixture into deep tissues, a modified culture procedure was employed before application of virus into kidney rudiments, based on an early study which shows that cold storage of isolated kidney rudiments in PBS retains the viability of the kidneys for several hours [23]. Unlike previous methods that involve direct addition of molecular agents into medium, isolated embryonic kidneys were incubated with transfection mix or virus in a low-serum medium at 4°C for 6–8 hours before being seeded in the culture vessel (Figure 1(a)).

### 3.2. Highly Transduction Efficiency and Penetration of scAAV

**Figure 2:**
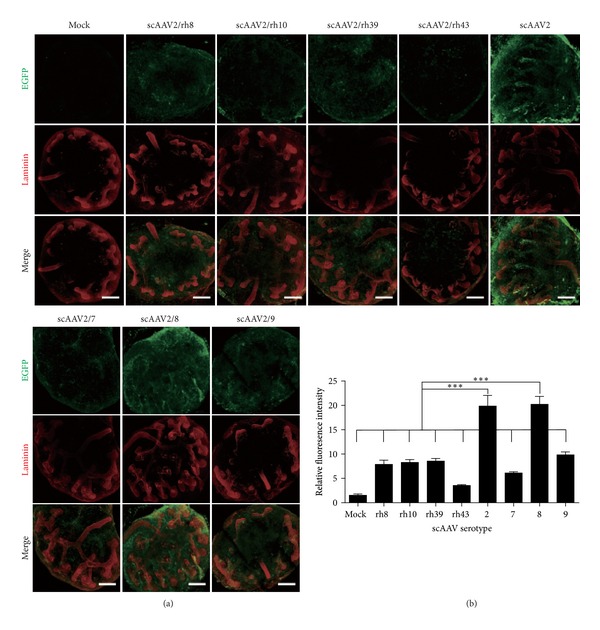
Transduction of embryonic kidney* in vitro* with different serotypes of scAAV. After incubation of the E12.5 kidney rudiments with different serotypes of scAAV at 4°C for 6 hours, the kidneys were cultured at 37°C for 24 hours. (a) EGFP (green) shows the gene expression of scAAV. The basement membrane marker laminin (red) shows both the UB and nephrons. Bar = 200 *μ*m. (b) The relative EGFP fluorescence intensity was measured with ImageJ. All serotypes were tested for three times, with 3 kidney rudiments at each time.

Although* Lentivirus* and adenovirus were widely employed for* in vivo* and* in vitro* application, like siRNA transfection, they are unevenly distributed and appear to be excluded from the cap mesenchyme (Figure S1), which is in line with published papers [11, 14]. scAAV is a promising vector which gained much attention for gene transfer and gene therapy in the last decades [24]. There are dozens of AAV serotypes that bear different transduction efficiency [25]. To determine which serotypes hold the best gene delivery efficiency in embryonic kidney, 8 different serotypes (2, 7, 8, 9, rh8, rh10, rh39, and rh43) of pseudotyped scAAVs with the same scAAV2-based genome (Figure 1(b)) were employed to transduct intact mouse embryonic kidney rudiments. After preincubation of E12.5 kidney rudiments with the virus at 4°C for 6–8 hours, kidneys were cultured in 37°C for 24 hours. Direct fluorescence imaging showed that EGFP expression of serotypes 2 and 8 was significantly higher than that of the others based upon fluorescence intensity (Figure 2). The most serious weakness of gene manipulation methods, such as siRNA transfection, microinjection and electroporation, protein transduction, and* Lentivirus* and adenovirus transduction, is that they are not efficient in the condensed MM cells. The data showed scAAV serotypes 2 and 8 exhibited highly transduction efficiency and can penetrate into the deep of kidney.

### 3.3. Different Tropisms of scAAV Serotypes 2 and 8

**Figure 3:**
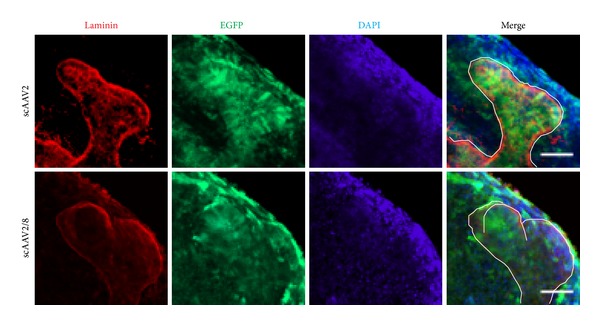
Tropism of scAAV serotypes 2 and 8 in cultured kidney. The tropism of scAAVs 2 and 2/8 was analyzed after 1-day culture. Laminin (red) was used to mark the UB which was outlined with white line. The experiment was tested for three times, with 6 kidney rudiments at each time. Scale bar is 50 *μ*m.

Different serotypes of AAV show different tropisms in mice [15, 26]. To find the best suitable serotype of scAAV for the study of kidney development, the reporter gene expression of scAAV serotypes 2 and 8 was checked in transducted kidney rudiments. Serotype 2 preferred to infect the ureteric bud (UB) cells compared with metanephric mesenchyme (MM) cells, while serotype 8 showed ubiquitous infection ability to UB and MM cells (around the UB tips) (Figure 3). Optical serial sections confirmed these findings (Figure S2), suggesting both scAAV serotypes 2 and 8 were effective gene delivery tools in cultured kidney.

### 3.4. Expression Kinetics of scAAV during Nephrogenesis

**Figure 4:**
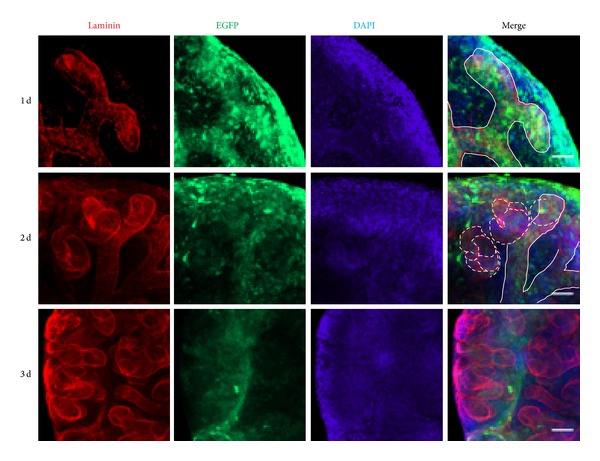
The expression kinetics of scAAV during nephrogenesis. The E12.5 kidney was transducted with scAAV2 and cultured for 1 to 3 days. Immunostaining of laminin (red) shows the UB (outlined with a white line) and newly formed nephrons (outlined with a white dashed line). The experiment was tested for three times, with 5 kidney rudiments at each time. Scale bar is 50 *μ*m.

Although scAAV genomes may persist within cells as episomes, low-frequency genomic integration was observed in previous publication [27, 28]. To further characterize scAAV transduction in cultured kidney rudiments, we examined the expression kinetics of scAAV during nephrogenesis. After preincubation with the widely used scAAV serotype 2, kidney rudiments were cultured for 1 to 3 days. At the first day, EGFP was detected in UB and MM. At day 2, EGFP was still expressed in UB, MM, and newly formed nephrons but absent from mature nephrons at day 3 (Figure 4). At day 3, EGFP were absent in all tubes except the root of ureteric bud (Figure S3). One possible reason is that scAAV was diluted out at day 3 due to fast cell division in MM cell during nephrogenesis. Taken together, the data showed that scAAV reporter gene EGFP was expressed in MM, UB, and immature nephrons and can be maintained longer in UB cells. These findings suggested that scAAV can be used for transient gene manipulation in the study of UB branching and nephrogenesis.

### 3.5. Knockdown of WT1 with scAAV Impairs Tubule Formation

**Figure 5:**
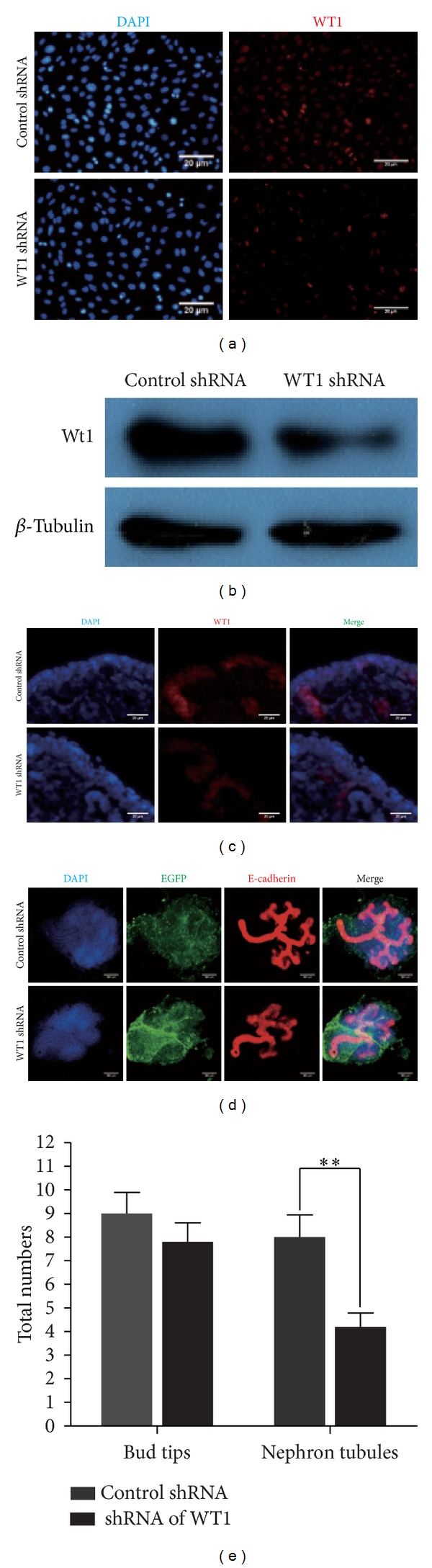
shRNA-mediated knockdown of WT1 with scAAV2/8 in mk3 cell and cultured kidney. (a) Immunostaining of mk3 (nuclear DAPI, blue, WT1, red) shows that Wt1 expression is repressed in most cells treated with Wt1 shRNA containing scAAV2/8 after 36 hours. (b) This repression was confirmed by western blot. (c) WT1 immunostaining (red) of cultured kidneys after 24 hours shows that WT1 expression is repressed in CM after Wt1 shRNA containing scAAV2/8 treatment. (d) Immunostaining of epithelial maker E-cadherin (red, showing both UB and newly formed nephron tubules, which were marked with a white arrowhead) in cultured kidney after 48 hours. The experiment was tested for three times, with 5 kidney rudiments at each time. (e) The total UB tips and nephron tubules were counted and levels of significance were determined using Student's* t*-test. Error bars represent standard errors of the mean. The asterisk indicated a significant difference at *P* < 0.01.

To validate the utility value of scAAV, we delivered shRNA of WT1 (Figure 1(b)) into cultured kidney with scAAV2/8. The knockdown efficiency of WT1 shRNA scAAV was confirmed in mK3 cell and cultured kidney by immunostaining and western blot (Figures 5(a)–5(c)). In line with published paper [11], after 2 days, WT1 shRNA scAAV treated kidneys show decreased WT1 expression in CM and decreased tubes formation (Figures 5(c)–5(e)). Ureteric bud-specific marker calbindin needs to be used in combination with E-cadherin to confirm whether nephrongensis is impaired. These data suggest that scAAV can deliver gene in CM and would be a promising tool for kidney development study.

## 4. Discussion

Compared with other virus vectors, scAAV holds many advantages: it is safe since no pathology of wild-type AAV serotype 2 was found till now; the diameter is 20–25 nm, smaller than that of adenovirus (90–100 nm) and* Lentivirus* (150–200 nm); and the delivered gene in self-complementary viral genome can be expressed faster than that of retrovirus and* Lentivirus*. As we know, the nephrogenesis progressed rapidly and the development time of the embryonic kidney is short. It is essential to express delivered gene in a fast and highly effective manner. Lentiviruses that infect both dividing and nondividing cells with high efficient genomic integration should be an effective tool for gene delivery in* ex vivo* cultured kidney. Unfortunately, lentiviruses are not capable of penetrating CM deeply. Up to now, scAAV is one of the best virus systems for cultured kidney.

More serotypes of scAAV and markers of kidney development should be tested in cultured kidney, as the cell tropism and transduction efficiency of scAAV serotype are different. Based on our findings, scAAV serotypes 2 and 8 hold the best transduction efficiency for cultured kidney in our study. For other* ex vivo* cultured organ models, scAAV also is a promising tool for organogenesis study. However, it needs to test which serotype of scAAV holds the best efficiency of gene delivery and cell tropism. One restriction of scAAV vector application in* ex vivo* cultured organs is its packaging capacity. While AAV generally delivers about 4.4 kb of unique transgene sequence, scAAV should be able to carry about 2.2 kb. It is sufficient for a great number of useful applications, using relatively small sizes of transgenes with simple promoters, and shRNA-based loss of function. Thus, our findings could lead to increased application of* ex vivo* organ cultures as a model in renal development.

## Supplementary Material

Figure S1. siRNA transfection, lentivirus and adenovirus transduction in cultured kidney After incubation of the E12.5 kidney rudiments with Cy3-siRNA-lipofectamine mixture, lentivirus and adenovirus at 4 °C for 6 hours, the kidneys were cultured at 37 °C for 24 hours. Base on the morphology, UB was outlined with a white line and CM was outlined with a white dash line. Bar=200 *μ*m
Fig.S2. Confocal optical serial sections of scAAV2 and 8 tropism Optical serially section of scAAV transducted kidney using confocal microscopy. The basement membrane was labelled with anti-laminin antibody (Red). Interlayer space is 2 *μ*m.
Fig.S3. The expression of scAAV2 in the root of ureteric bud. A. Diagram of the ureteric bud tree. B.The E12.5 kidney was cultured 3 days after scAAVs treatment, Anti-laminin immunostaining shows the root of ureteric bud (outlined with a white line) and newly formed nephrons (outlined with a dashed line). Scale bar is 50 *μ*m.
