# Supplementary material for: Highly Effective Ex Vivo Gene Manipulation to Study Kidney Development Using Self-Complementary Adenoassociated Viruses
Source: ScientificWorldJournal. 2014 Jul 14;2014:682189. doi: 10.1155/2014/682189 (PMC4123690; doi:10.1155/2014/682189)
Supplement: Supplementary file 1 — Figure S1. siRNA transfection, lentivirus and adenovirus transduction in cultured kidney After incubation of the E12.5 kidney rudiments with Cy3-siRNA-lipofectamine mixture, lentivirus and adenovirus at 4 °C for 6 hours, the kidneys were cultured at 37 °C for 24 hours. Base on the morphology, UB was outlined with a white line and CM was outlined with a white dash line. Bar=200 μm Fig.S2. Confocal optical serial sections of scAAV2 and 8 tropism Optical serially section of scAAV transducted kidney using confocal microscopy. The basement membrane was labelled with anti-laminin antibody (Red). Interlayer space is 2 μm. Fig.S3. The expression of scAAV2 in the root of ureteric bud. A. Diagram of the ureteric bud tree. B.The E12.5 kidney was cultured 3 days after scAAVs treatment, Anti-laminin immunostaining shows the root of ureteric bud (outlined with a white line) and newly formed nephrons (outlined with a dashed line). Scale bar is 50 μm. [file 682189.f1.pdf]

Supplementary data

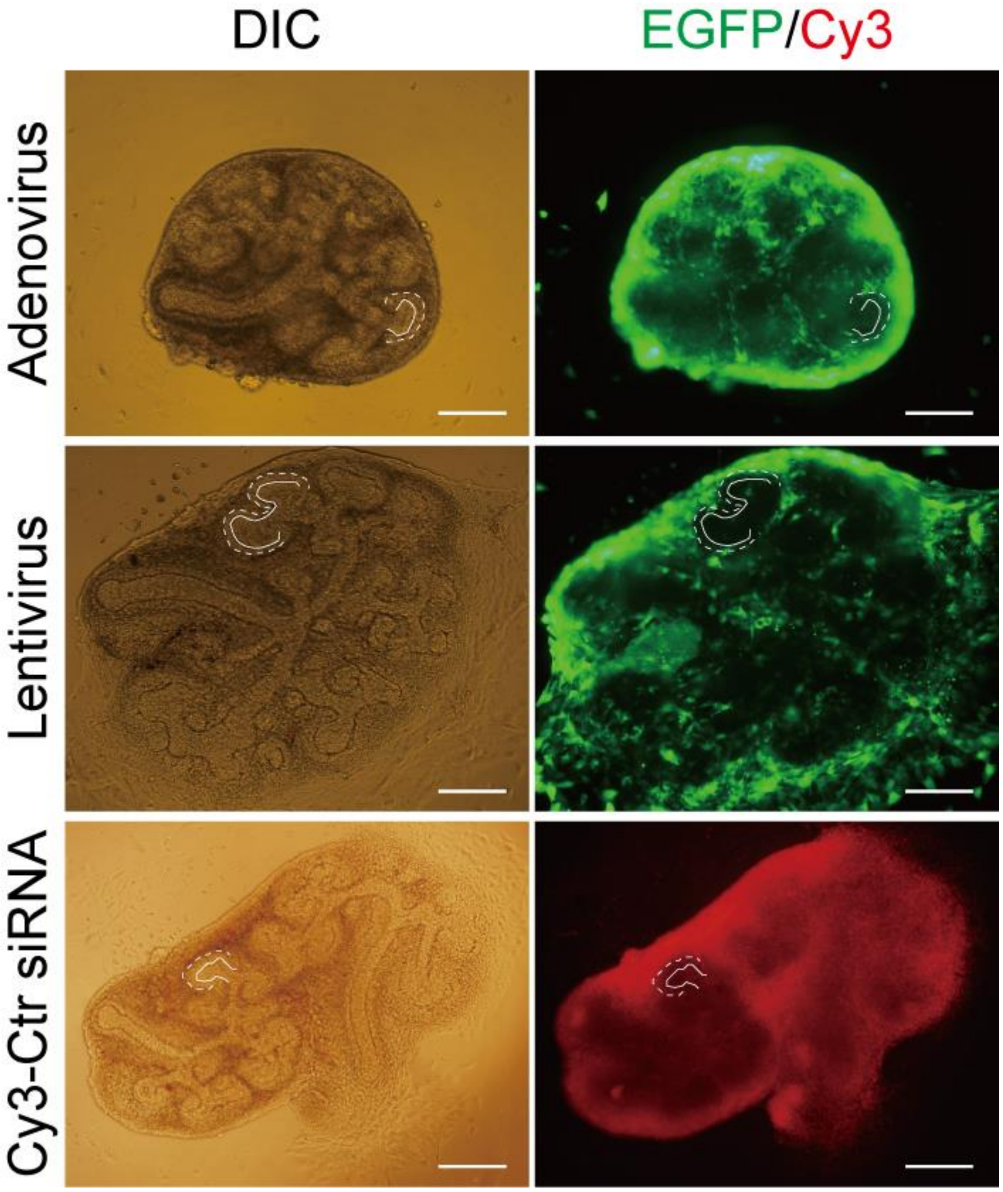

Figure S1. siRNA transfection, lentivirus and adenovirus transduction in cultured kidney

After incubation of the E12.5 kidney rudiments with Cy3-siRNA-lipofectamine mixture, lentivirus and adenovirus at 4℃ for 6 hours, the kidneys were cultured at 37℃ for 24 hours. Base on the morphology, UB was outlined with a white line and CM was outlined with a white dash line. Bar=200μm

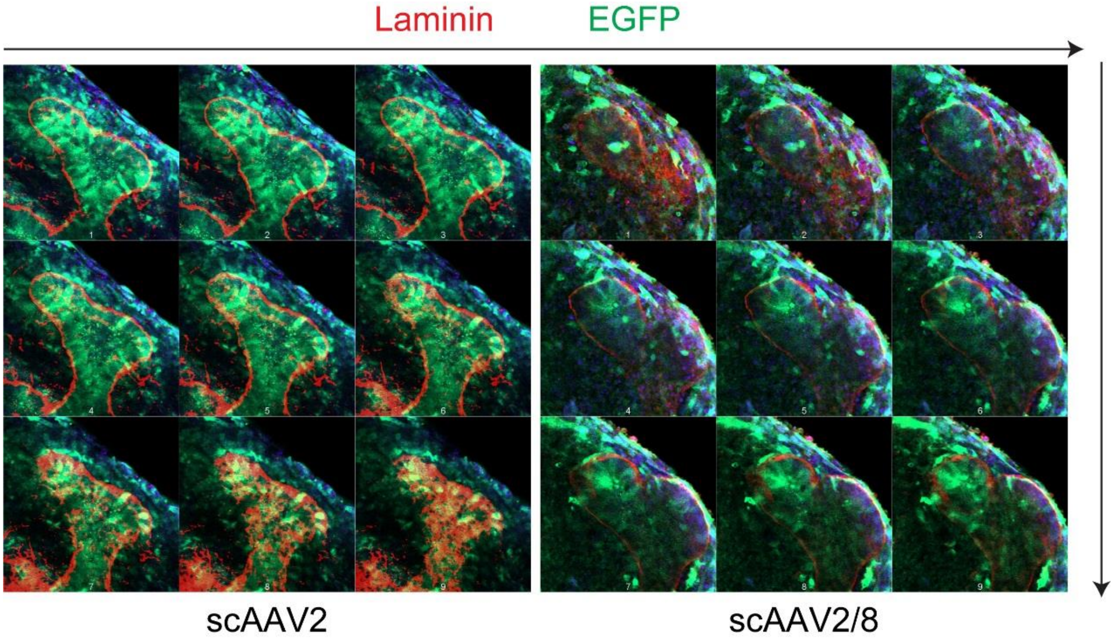

Fig.S2. Confocal optical serial sections of scAAV2 and 8 tropism

Optical serially section of scAAV transducted kidney using confocal microscopy. The basement membrane was labelled with anti-laminin antibody (Red). Interlayer space is 2 µm.

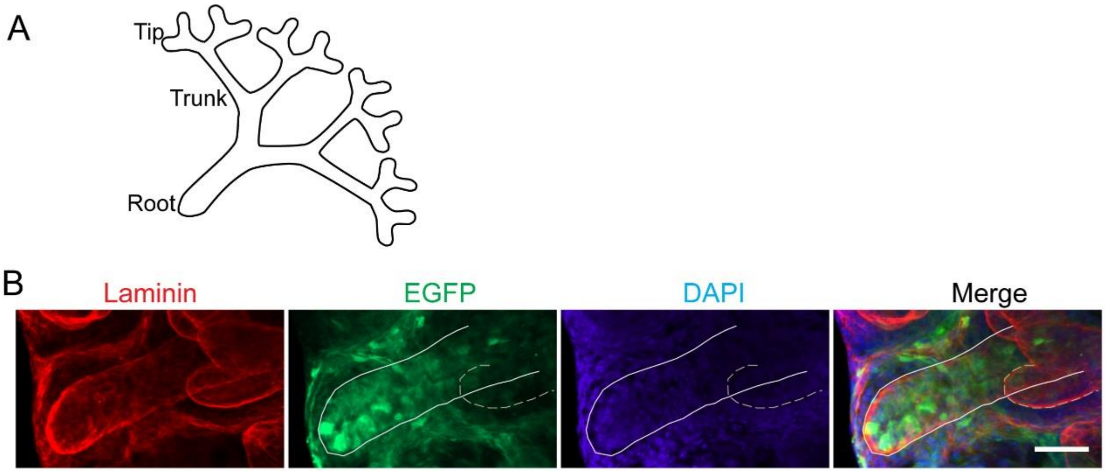

Fig.S3. The expression of scAAV2 in the root of ureteric bud.

A. Diagram of the ureteric bud tree. B.The E12.5 kidney was cultured 3 days after scAAVs treatment, Anti-laminin immunostaining shows the root of ureteric bud (outlined with a white line) and newly formed nephrons (outlined with a dashed line). Scale bar is 50µm
